# Modelling Management Practices in Viticulture while Considering Resource Limitations: The Dhivine Model

**DOI:** 10.1371/journal.pone.0151952

**Published:** 2016-03-18

**Authors:** Roger Martin-Clouaire, Jean-Pierre Rellier, Nakié Paré, Marc Voltz, Anne Biarnès

**Affiliations:** 1 INRA, UR 875 Mathématiques et Informatique Appliquées de Toulouse, Castanet Tolosan, France; 2 CAPNODIS, Paris, France; 3 INRA, UMR LISAH, Montpellier, France; 4 IRD, UMR LISAH, Montpellier, France; Université Toulouse 1 Capitole, FRANCE

## Abstract

Many farming-system studies have investigated the design and evaluation of crop-management practices with respect to economic performance and reduction in environmental impacts. In contrast, little research has been devoted to analysing these practices in terms of matching the recurrent context-dependent demand for resources (labour in particular) with those available on the farm. This paper presents Dhivine, a simulation model of operational management of grape production at the vineyard scale. Particular attention focuses on representing a flexible plan, which organises activities temporally, the resources available to the vineyard manager and the process of scheduling and executing the activities. The model relies on a generic production-system ontology used in several agricultural production domains. The types of investigations that the model supports are briefly illustrated. The enhanced realism of the production-management situations simulated makes it possible to examine and understand properties of resource-constrained work-organisation strategies and possibilities for improving them.

## 1. Introduction

Agricultural systems are complex and associated with technologies and crop-management practices that cause severe environmental damage, such as soil erosion, water and air pollution, and increased greenhouse gas emissions. There is thus an overwhelming consensus [1–3] that new management procedures are needed to move towards environmentally friendly management behaviour without jeopardising economic efficiency. The European Water Framework Directive [4] established groundwater quality standards along with an agenda for European countries to take action towards eliminating or limiting introduction of pollutants into groundwater. Vineyards in France are especially concerned, given the large amount of pesticides generally used on them and the water pollution that ensues [5]. Nevertheless, no significant improvement in water pollution in vineyard areas has been observed for more than a decade [6]. For technical, economic and social reasons, it is not feasible to require all vineyards in France to move from a traditional farming system to an organic system, which would certainly solve part of the pollution problem while potentially creating new pressure points. A more realistic initial step is to identify which crop-management practices could be improved to decrease water pollution in existing vineyards.

Temporal and spatial distributions of soil treatments and spraying methods at farm and catchment scales are of paramount importance because they, combined with rainfall events, can lead to pollution peaks (e.g. [7]). Developing cleaner crop-management strategies is not an easy task. It requires precisely linking water-contamination dynamics to vineyard management-practices to detect those with the largest impact. Unfortunately, few data exist about the frequency of potential pollution peaks because in many countries farmers are under no obligation to register their practices. Moreover, pesticide-transfer models poorly explain the spatial and temporal combination of agricultural practices and their effects (see [8] for a review).

Another difficulty in changing crop-management practices stems from the specific material constraints under which the farm operates. Any change incompatible with these constraints cannot be adopted. Several new environmentally friendly management practices were suggested by water and/or agricultural state agencies in France. These were rarely adopted by farmers, however, because they did not consider available resources (e.g. labour and equipment) and work organisation (see [9, 10] for examples concerning annual crop production in northern France).

Economic and environmental risks resulting from poor work organisation and crop-management practices are quite serious. Operations may not achieve the expected outcomes or may cause undesired effects, especially if performed at the wrong time and under inappropriate circumstances. While many recognise the importance of developing a deeper understanding of farmers’ management behaviours (see conclusions of [11] and [12]), the subject is rarely an object of scientific investigation, to the best of our knowledge. In particular, little research has been devoted to feasibility analysis of management practices in terms of matching the recurrent context-dependent demand for resources with those available on the farm. In agreement with [13], we believe that a modelling approach that simulates work activities in their temporal and spatial dimensions at the farm scale can help greatly to identify problematic situations and explain their occurrence in relation to work organisation (which supports the scheduling of crop-management activities and allocation of resources to perform them) and management’s responsiveness to uncontrollable events (e.g. weather, infestations). Such approaches may become a way for researchers and practitioners to conduct virtual experiments of work-organisation strategies and identify shortcomings and possible improvements.

This paper presents such a simulation approach for vineyards, with particular emphasis on realistic modelling of dynamic scheduling of work activities and resource allocation to these activities (labour and machinery). The need goes beyond simple extrapolation of activity management from the field to the farm scale. The farmer’s numerous activities interact, compete for the same resources and require trade-offs to deal with the heterogeneity of the production system. Therefore, planned and adaptive coordination of the activities becomes central in farm production to cope with uncertainty (weather, essentially) and resource limitations. Resource allocation is a repetitive decision-making task and concerns a context-dependent set of activities, the execution of each requiring resources with various capabilities and limited availability. This creates contention and the need to determine an optimal subset to allocate and an optimal combination of resources.

There are a wide variety of approaches for whole-farm models [14]. All integrate management and biophysical considerations, but with different emphasis depending on their primary purpose, such as policy or innovation assessment [15], analysis of climate-change adaptation or mitigation [16–17], and participatory design of farming system adaption to new conditions[18–19]. The models also differ in their methodological frameworks. In an optimisation setting, models must be simple (often static) to preserve computational tractability. With simulation, more realistic representation of decision-making is possible as long as the level of granularity remains compatible with the available data and their usability by analysts interpreting the results.

Most such simulation models have a management component that is represented by a set of simple decision rules that implement a type of reactive and unconstrained management behaviour. They primarily focus on biophysical processes and are rarely concerned with resource-related considerations beyond rough economic accounting of the resource consumed. Not considering resource constraints properly in the analysis may overestimate benefits of operations [14]. Except for models of single-resource management, such as of irrigation water [20, 21], we know of only two simulation models in agriculture that deal explicitly with the problem of dynamically allocating resources required by activities to accomplish a production plan. The model OTELO [22, 23], developed in the late 1980s, represents interactions between farm activities, their implementation, and a limited supply of resources (labour, machinery). It was used to analyse peak periods in the cropping calendar when harvesting overlaps with sowing, thus creating simultaneous demand for material and labour. Unfortunately OTELO’s language does not support introduction of new types of allocation constraints besides those hard-coded into the model. Moreover the ability to express biophysical conditions of activities’ feasibility or pertinence was too limited to apply it to vineyards. Viticulture management [24] is a complex year-round process that involves many technical operations which are performed on heterogeneous plots with specific management requirements. The other model, APSFarm [25, 18], was recently developed to simulate the dynamic use of land for different crops or livestock production. APSFarm includes the restriction of resources such as labour, irrigation water, and machinery for operations that correspond to a change in land use (e.g. from fallow to wheat). The APSFarm decision-making approach was not designed to support analysis of resource-allocation problems, i.e. the permanent trade-off farmers make in scheduling daily activities and managing resources whose availability varies over time.

Resource allocation is, in essence, a combinatorial problem. The problem must be solved dynamically because the activities requiring resources are not necessarily known in advance, the feasibility conditions for meeting the need are beyond the manager's control, and the availability of resources is uncertain. The models used in agriculture generate convenient simplifications by considering single-resource problems (assuming no restrictions occur in the other resources) or by assuming that activities are instantaneous or not interrupted (which would require allocation to be recalculated at each interruption).

Unlike in agriculture, work organisation involving plan-based decision-making and resource-limitation problems has received much attention in systems engineering [26, 27] and management science in the business, logistics and manufacturing domains [28]. Research on workflow [29, 30] has produced concepts and languages that can be used to describe organisational policies and procedures. Much of this conceptualisation is generic and therefore applicable to agricultural production management.

This paper presents and evaluates an original model for simulating temporal and spatial patterns of crop-management practices in a vineyard. The underlying modelling approach rests on the central concept of activity within a workflow. It is embedded in a simulation framework, called DIESE [31], that provides a dedicated inferential mechanism that simulates the decision-making process involved in production management, including work scheduling and resource allocation. The simulation engine was designed to model the management process in a dynamic environment requiring responsive behaviour to a situation as it occurs.

The model described here, referred to as Dhivine [32], provides ready-to-use knowledge components that can be assembled and customised to simulate multiple vineyard systems simultaneously. It specifies in detail the logic underlying the temporal organisation of vineyard-production activities and the resource capacity (resource pool) of the vineyard manager. The intended timing of activities is specified flexibly and almost always hinges on a suite of dynamic factors that include the biophysical state of the system, occurrence of external events, progress in implementing the management strategy, and resource availability. The model simulates the process by which these activities are dynamically scheduled and performed as a function of current conditions. The scope of the paper is restricted to the simulated management of a single vineyard.

## 2. Fundamental Modelling Concepts

Dhivine was developed using the DIESE object-oriented framework [31], which is based on a production-system ontology [33]. Such an application-domain ontology [34] is an explicit and declarative description of the concepts in the domain, the properties of these concepts and the constraints on these properties. Object-modelling technology for model analysis and design is especially suitable for declarative modelling of complex systems with many interacting components, whose semantics are described in an unambiguous and machine-processable form. The ontology provides a common vocabulary and enables repeated use of pre-formalised concepts and templates; thus, it serves as a conceptual meta-model for the modelling framework. These concepts and templates, implemented as classes, can then be specialised (by creating subclasses) and mapped into an executable model interpreted with a discrete-event simulation engine. See [32, 35–38] for examples of simulation models using DIESE for different agricultural systems.

Because a production system evolves over time, DIESE ontology is based on three fundamental concepts: *entity*, *process* and *event*, which represent the structural, functional and dynamic aspects of a system, respectively. An entity describes a type of material or immaterial item, such as an object (e.g. a plot or a tractor), an aggregate object (e.g. a production system), a location, a goal or a constraint. Each entity is associated with specific properties that can be attributes (a feature taking a numerical or qualitative value), relationships of containment (e.g. “is composed of”, “is part of”), subclass relationships (particularisation), association relationships (e.g. spatial connectivity such as “adjacent to”), and methods (procedural knowledge associated with the entity). The state of a system at a given moment is the value of the properties of the entities it comprises. A process specifies a change in or behaviour of part of a system (i.e. of some of the entities it comprises). A process is either instantaneous (e.g. issuing an instruction) or extends over a period (e.g. a growth process). The main property of a process is its functional attribute, which codes changes to be made to the system when the process runs. A process causes or initiates a change in state only when a particular event occurs in the environment of the part of the system concerned. Events take place at specific times and, thus, convey the temporality of process triggers.

For managerial aspects, the fundamental concept is an *activity*, a specialised form of an entity. In its simplest form, an activity corresponds to a single unit of work (a “primitive activity”). It designates something to be done to a particular biophysical object or location (e.g. a plot) by a labour resource (one or several workers) and, possibly, using or consuming other material resources (e.g. a tractor). A primitive activity has local opening and closing conditions that are necessary for the activity to enter the status *open* (i.e. eligible for execution) or *closed* (i.e. eligible to be stopped). These conditions are defined by time windows and/or predicates (Boolean functions) referring to biophysical states or indicators. The “something-to-be-done” component of a primitive activity (an “operation”) is an intentional transformation of the controlled system (e.g. herbicide spraying). Like processes, operations can be considered instantaneous (closing a tank) or to take some time (spraying). In the latter case, the operation is characterised by a speed at which it is executed (e.g. number of items or amount of area which can be processed per unit of time and per unit of labour). Execution of an operation is constrained by feasibility conditions that are linked to the biophysical system state. Execution of an operation requires the primitive activity that contains it to have the status *open*.

Activities can be further constrained by using programming constructs with evocative names such as *Before*, *Meeting*, *Iterate*, *And*, and *Optional*, which enable specification of temporal ordering, iteration, aggregation, and optional execution of primitive activities by creating compound (“non-primitive”) activities. All activities except one, the “plan”, are connected through these constructs. The plan, the top-level activity, is the only one not involved in a higher-level activity through a programming construct. To be executable, each activity must satisfy (i) proper opening conditions and (ii) interdependency conditions between all activities interconnected with the previously mentioned constructs. These conditions determine their dynamic status. For example, in the activity *Before(A*, *B)*, activity *B* can be *open* between dates 10 and 20 but will become *open* within this interval only after A has become *closed*. These conditions and the meaning of the constructs make it possible to confer some flexibility to the plan, which plays a guiding rather than a prescribing role at the time of design. The primitive activities to be executed are determined dynamically as a function of the current internal situation (biophysical state and already-executed activities) and external events. Another source of flexibility supported by the management-plan representation comes from abstract specification of the set of entities (e.g. plots) processed by an operation. These entities are contextually selected at runtime.

A plan may encounter situations in which its initial intention goes beyond its bounds as particular events occur, e.g. a lasting drought. The rule that associates such a condition with the specification of changes that should be made to a nominal plan is called a conditional adjustment. The triggering condition is either a calendar condition that becomes true when a specific date is reached, or a state-related condition that becomes true when the current circumstances match this condition. The adjustment can be any change to the nominal plan, such as the removal or insertion of activities. It can also affect the resources used in some activities. This allows management to respond rapidly to cope with unexpected fluctuations in the external environment.

**Fig 1:**
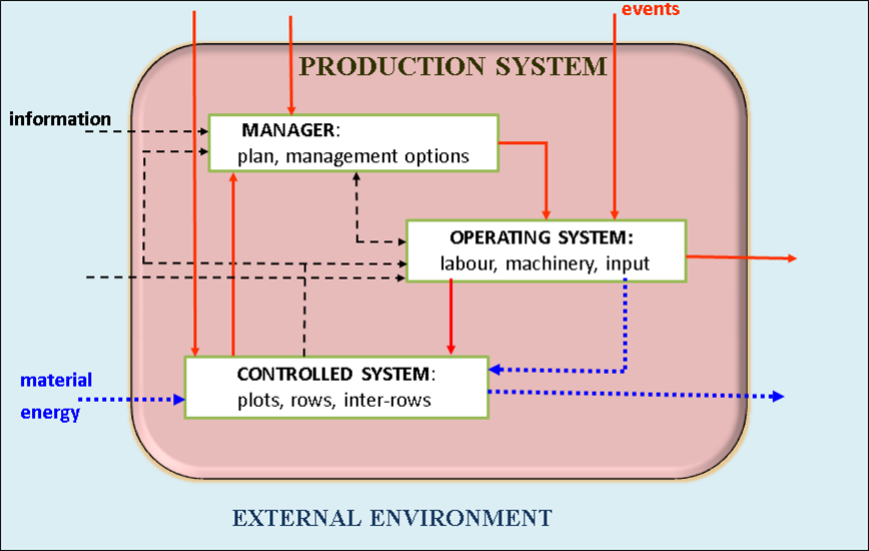
Diagram of a grape production system. Arrows represent flows of events (red), information (dashed black), and matter or energy (dotted blue).

**Fig 2:**
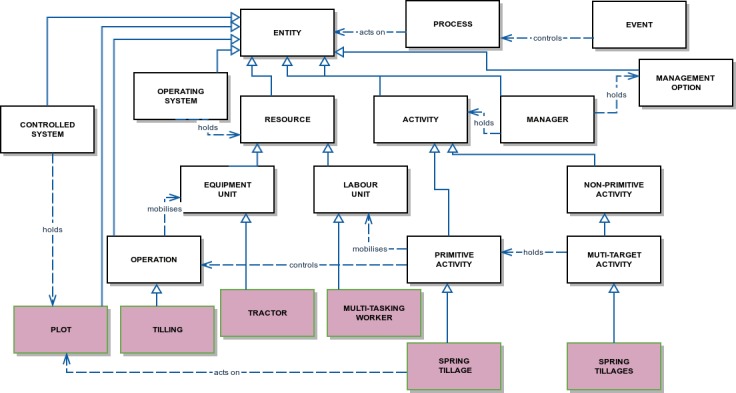
Main modelling concepts and their connections. Boxes represent classes from the DIESE framework (white) and those specific to Dhivine (shaded). Solid arrows represent particularisation relationships and dashed arrows association relationships.

Conceptually, an agricultural production system (Fig 1) is an entity located in and influenced by the external environment (e.g. the climatic and pest hazard context). It can be divided into three interacting subsystems: the decision-making system (e.g. the vineyard manager), the operating system and the controlled system, which are active entities in the sense that each is the repository of processes and has inputs, outputs and an agenda of events. The manager has a management strategy, which in Dhivine is a flexible user-defined plan accompanied by management options. The operating system is the repository of resources at the disposal of the manager to execute the intended primitive activities. The controlled or biophysical system is composed of biophysical entities whose change in state is made explicit in state-transition processes. The inputs are material inputs (e.g. pesticides provided by the operating system) and energy provided either by the external environment or the operating system. The ontology and the DIESE framework that implements it allow users to represent a set of independent production systems (e.g. vineyards in a catchment) and simulate them simultaneously. Interactions between them occur through incoming and outgoing events and physical material. Fig 2 provides a synoptic view of the main concepts (white boxes) and some of their particularisation (shaded boxes) for the Dhivine model presented in the next section.

## 3. Representation of a Grape-Production System in Dhivine

The knowledge incorporated into Dhivine (the three subsystems of Fig 1) comes from a survey of 54 vineyards in the Montpellier region of southern France [32]. In this paper, Dhivine is applied to the exemplifying vineyard “MG11”.

### 3.1. The controlled system and its environment

Because grapevines are the only production of most vineyard managers in southern France, the controlled system has only one component: a vineyard consisting of a set of vine plots, each identified by its longitude and latitude and characterised by alternating rows and inter-rows. The regular spacing of vines is described by the distance between two rows (row spacing) and the distance between two adjacent vine stocks in a row (vine spacing). Each plot contains a single grape variety characterised by its earliness and shoot vigour.

The only vine-related process used in Dhivine is the plant life cycle, which functions according to phenological stages. Regional databases provide the dates when stages are reached for a particular grape variety and a particular year.

The vineyard is subject to weather, pests, and disease (the external environment in Fig 1). Weather data for a plot (currently, only hourly rainfall) come from the nearest weather station, identified by latitude and longitude. Pest and disease pressure is assumed to be the same throughout the vineyard, and knowledge of this pressure comes from regional agricultural warning services.

### 3.2. The operating system

Dhivine deals with two types of resources: machinery and labour units. The resources are tied to general or situational restrictions on their use. Labour units are distinguished by their status (standard worker or chief operating officer) and skills. A skill defines a class of labour units and is associated with a set of possible tasks (e.g. multi-tasks, tractor driving, pruning). For example, in the MG11 vineyard, the available labour units are represented by two instances of the class of multi-tasking workers, one of them with the status of chief operating officer, and by two instances of the class of standard pruning workers. Employment of labour units is modelled by an annual timetable that depends on their status and skills. The timetable includes seasonal variability in both working days during the week and working hours during the day. It includes a calendar of workers’ days off. Consequently, the availability of a labour unit is constrained by its annual timetable. There is no such constraint for machinery resources.

### 3.3. The management system

#### 3.3.1 The concept of a multi-target activity

In many crop production systems, work is primarily structured around interlaced plot-specific plans, each specifying the intended temporal organisation of the operations performed on a given spatial entity. The emphasis is on temporal rather than spatial organisation of operations. To represent management strategies that are more balanced in temporal and spatial dimensions, Dhivine uses a special kind of compound activity called a *multi-target activity* (MT-activity). An MT-activity specifies application of a cultural operation (e.g. tilling) to each plot of a well-defined set. In other words, an MT-activity supports a mode of work organisation focused on a set of spatial entities (i.e. plots in Dhivine) processed by the same single type of operation, each application constituting a primitive activity. The need for such management specification was first mentioned by Aubry et al. [39] for wheat management on an arable farm: "The relevant units for designing management are, rather, sets of fields to which it has been decided to apply the same timing and/or the same mode of operation."

In addition to its operation and feasibility conditions, an MT-activity is characterised by (a) its speed, which depends on resources; (b) the implementation mode (in sequence or in parallel); (c) the set of plots and their order of visitation; and (d) the opening and closing conditions. In Dhivine, MT-activities are given a name that ends with an “s” (e.g. *SpringTillages*) and when there is no ambiguity, they are designated by referring to their underlying operation. When two MT-activities compete for resources, the priorities of these activities and the underlying operations are invoked (see § e below).

**a–Resources, speed and feasibility conditions:** Because it concerns a cultural operation, an MT-activity mobilises sufficient equipment and labour to perform the operation. It is also constrained by feasibility conditions and the speed of the operation. See Table 1 for examples of the resource requirements, speed and feasibility conditions involved in the management of MG11. It is assumed that feasibility conditions of manual operations (e.g. pruning) are always satisfied. For motorised operations, feasibility conditions concern soil trafficability and, for tillage, workability. In Dhivine, trafficability and workability depend on cumulative rainfall in the 7 days preceding the day of the intended operation.

**Table 1:** Multi-target (MT) activities and their characteristics in the example MG11 vineyard.

Operation of the MT-activity	Equipment	Labour requirement	Nominal speed	Proportion of inter-rows visited	Feasibility conditions
Trimming	1 clipper + 1 of 3 available tractors	1 of 2 available multi-tasking workers	6.0 km/h	0.5	Trafficability: R[j—7; j] < 60 mm
Chemical weeding and chemical trunk shoot removal	1 tank + 1 handheld gun + 1 of 3 tractors	1 of 2 multi-tasking workers	4.0 km/h	1	Trafficability: R[j—7; j] < 60 mm
Fertilisation	1 spreader + 1 of 3 tractors	1 of 2 multi-tasking workers	6.0 km/h	1	Trafficability: R[j—7; j] < 60 mm
Pesticide spraying	2 x (1 air assisted sprayer + 1 of 3 tractors)	2x1 multi-tasking worker	6.0 km/h	0.5	Trafficability: R[j—7; j] < 60 mm
Pruning	-	2 pruning-workers + the max number of 2 multi-tasking workers	75 stocks/h	-	-
Shoot thinning	2 x (1 grinder + 1 of 3 tractors)	2x1 multi-tasking worker	5.0 km/h	0.5	Trafficability: R[j—7; j] < 60 mm
Tillage	2 x (1 tine cultivator + 1 of 3 tractors)	2x1 multi-tasking worker	4.0 km/h	1	Workability: R[j—7; j] < 40 mm
Trellising	-	2 multi-tasking workers	600 stocks/h	-	-

R[d1; d2] = cumulative rainfall between d1 and d2; j = current day. The proportion of inter-rows visited equals 0.5 when the tractor has to pass on one out of two inter-rows to perform the operation and 1 when all inter-rows need be visited.

The speed of performing an MT-activity depends on several factors: the nominal speed (NS) of the underlying operation, the unitary speed (US) and the number of workers involved. In Dhivine, NS is expressed in vinestocks/h/person for manual operations and in km/h/person (tractor driver) for motorised operations (Table 1). The unitary speed US is expressed in ha/h/person and its value depends on characteristics of the plots as follows:

- Manual operations: US = (IS × IR × NS) / 10^8^, with IS (in cm) the inter-stock distance and IR (in cm) the inter-row distance.
- Motorised operations: US = (IR × NS) / (1200 × P), with P the proportion of inter-rows the tractors have to visit to perform the operation. The normalising factor 1200 includes a 20% reduction in speed to account for time lost manoeuvring the tractor. The nominal speed NS is assigned a value that can take into account the plot slope or other local considerations.

The speed of performing an MT-activity (ha/h) is the product of US and the number of workers. The speed can vary over time because the number of workers can change.

**b—Implementation mode of multi-target activities:** Two types of MT-activities are modelled in Dhivine. They correspond to two modes of implementation of underlying primitive activities observed in vineyards. An MT-activity is "parallel" when the type of operation, the overall resource situation, or any manager preference justifies that two or more plots can be processed simultaneously (possibly with partial overlap of execution periods) in any order. When operations are performed on each plot sequentially without overlap, the MT-activity is called "series".

For the MG11 vineyard, resource capacity is a priori compatible with simultaneous tillage of two plots. This is also valid for shoot-grinding and pesticide-spraying activities. They are declared as parallel MT-activities and are effectively executed simultaneously if the required resources are available at execution time. The other motorised MT-activities (i.e. that involve operations of fertilisation, chemical weeding, trimming, chemical trunk shoot removal) are performed sequentially, as are the manual activities (i.e. pruning, trellising).

**c—Dynamic selection of plots:** The manager makes a responsive activity schedule based on information she/he has on the progress of activities, equipment and labour availability, and the short-term weather forecast, which determines the feasibility of cultivation operations. For a given MT-activity, the plots to process are identified twice a day by a procedure specific to this activity. The general principle of this procedure is as follows. When opening a MT-activity, an initial list of plots to process is established. At each update, the fully processed plots are removed from the list and those in progress are kept on the list. The plots not yet visited are returned to the set of candidates from which a selection is made. Depending on the current biophysical, organisational and weather context, these plots are reselected or replaced by others. For some MT-activities, not all plots are necessarily processed. For example, bush-trained plots will not be selected for trellising activity. The plots to be processed are scheduled by respecting some constraints; for example, a plot cannot be included on the list for shoot grinding if it is not first pruned. Similarly, in the spring, a plot is not included in a tillage MT-activity if the number of days since its tillage lies below a certain threshold. For some MT-activities, not all plots are necessarily processed. For example, bush-trained plots will not be selected for trellising activity.

**d—Temporal constraints:** Each MT-activity has opening and closing predicates (as primitive activities) to determine the point at which the activity can begin and the point at which it can no longer be performed. In the MG11 vineyard (Table 2), for example, predicates refer to calendar dates (descriptor 1), biophysical states (descriptor 11), progress of other MT-activities (descriptor 19) or a combination of conditions (descriptor 4). An MT-activity is usually closed when all candidate plots concerned with this activity have been processed. Closing predicates for MG11 are specified only for activities that are exceptions to this rule; for example, trunk shoot removal is stopped on 15 June even if all the concerned plots have not been processed (descriptor 21).

**Table 2:** Opening (OP) and closing (CP) predicates and other descriptors used in the predicates.

#	Descriptor name	Value
*1*	*Autumn tillage OP*	If j ≥ 20 Oct
*2*	*Autumn tillage CP*	If j ≥ 31 Dec
*3*	*Late winter tillage OP*	If j ≥ 15 Feb and R[01 Nov; 15 Feb] > 200 mm; else if j ≥ 15 Mar
*4*	*Spring tillage OP*	If {date of last tillage < j-30 and R[date of last tillage; j] > 40 mm on a vineyard area > *Spring tillage OP scop*e} or {date of last tillage = j-45}. For the latter, only one iteration is performed.
*5*	*Spring tillage OP scope*	5 ha
*6*	*Spring tillage iteration CP*	If j ≥ 01 Jul
*7*	*Chemical weeding OP*	If j ≥ 25 Feb and *Thinning* and *Pruning* are finished
*8*	*Spring chemical weeding OP*	false (i.e. no spring chemical weeding)
9	*Early variety blueprint*	Chardonnay
10	*Late variety blueprint*	Carignan
11	*Anti powdery mildew pesticide regular spraying OP*	If the late variety blueprint has reached the ‘5–6 leaves’ stage
12	*Anti powdery mildew pesticide regular spraying CP*	If the late variety blueprint has reached the ‘berry-touch’ stage
13	*Anti downy mildew pesticide regular spraying OP*	If the late variety blueprint has reached the ‘5–6 leaves’ stage and j corresponds to a day in the pest and disease alert file with a downy mildew alert and if R[j; j+3] ≥ 10 mm
14	*No-pressure anti downy mildew pesticide regular spraying CP*	If the late variety blueprint has reached the ‘berry-touch’ stage
15	*High-pressure anti downy mildew pesticide regular spraying CP*	If the late variety blueprint has reached the ‘early-ripening’ stage
16	*Anti powdery and downy mildew pesticide regular spraying frequency*	14 days
17	*Anti powdery mildew extra pesticide spraying OP*	If j corresponds to a day in the pest and disease alert file with a powdery mildew alert and the next regular spraying is programmed within 6 to 7 days
18	*Fertilisation OP*	If j ≥ 20 Oct
19	*Pruning OP*	If j ≥ 01 Nov
20	*Shoot grinding OP*	If at least 12 ha have been pruned
21	*Trunk shoot removal OP*	If j ≥ 20 Apr
22	*Trunk shoot removal CP*	If all the plots have been processed or j ≥ 15 Jun
23	*Trellising OP*	If the early variety blueprint has reached the ‘early flowering’ stage
24	*Trimming OP*	If j ≥ 20 May

j = current day; R[d1; d2] = cumulative rainfall between d1 and d2

**e—Priority degrees:** MT-activities are assigned degrees of priority for resource allocation as well as degrees of execution priority because they are likely to require the same equipment and/or labour at the same time. These degrees of priority are numerical and are exploited by the general simulation algorithm (see Subsection 4.2) to mimic the way vineyard managers manage situations of competition for resources.

When the available labour allows for several simultaneous MT-activities, degrees of priority for resource allocation are used to prevent activities that do not require specialised labour from mobilising a given resource at the expense of another activity that must have it. For example, they prevent manual MT-activities from mobilising tractor drivers at the expense of motorised MT-activities. For MG11, to ensure that pruning does not mobilise workers who can drive tractors (i.e. multi-tasking workers), which would potentially preclude shoot grinding and late-winter tillage, these last two MT-activities are given higher resource-allocation priorities than pruning.

When several sets of activities compete for execution, the degrees of execution priority are used to select the set of activities performed. For example, in spring, the activities of the MG11 manager have the following priority: pesticide spraying > trellising > trimming > trunk shoot removal > spring tillage.

#### 3.3.2. Management strategy

**Fig 3:**
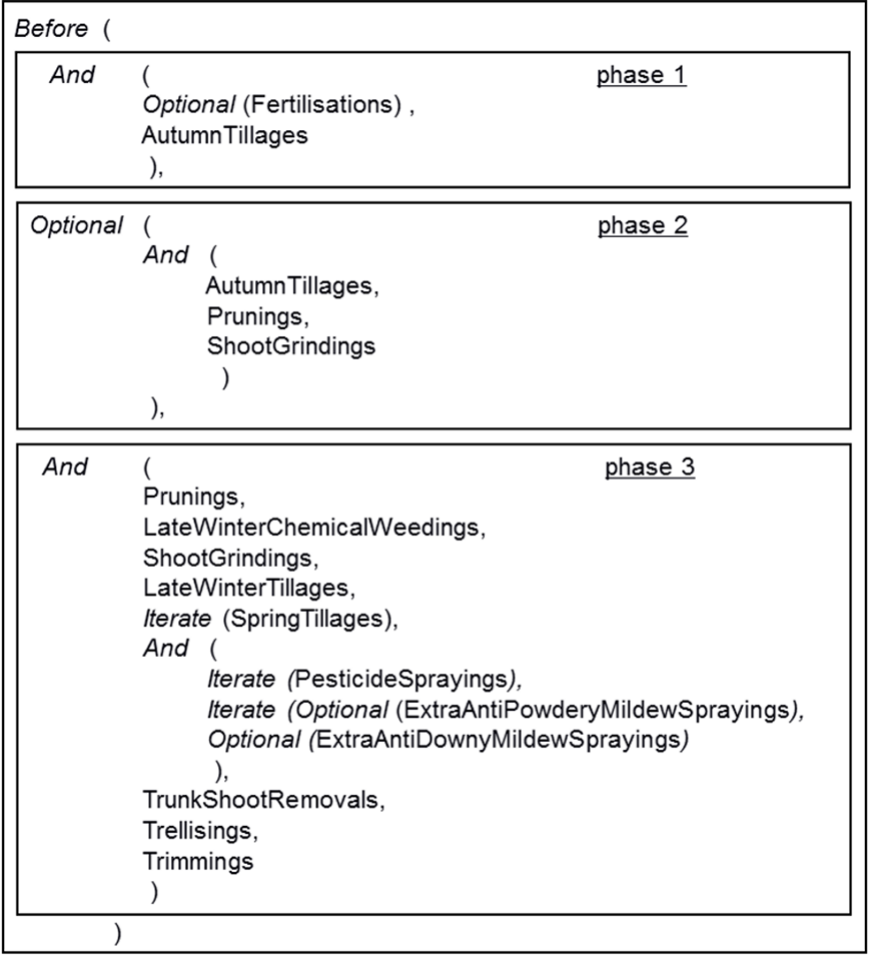
Plan used for the example MG11 vineyard.

In Dhivine, vineyard management follows an annual crop cycle. The manager’s management strategy is basically composed of (i) a general plan that loosely organises all possible MT-activities and (ii) management options that refine the plan with implementation details about the MT-activities. For example, the plan used for MG11 is organised in a chronological succession of three phases, each essentially consisting of an unordered list of MT-activities (Fig 3). Knowledge of the transitions between phases is contained in predicates that define the opening and closing conditions of the phases.

Some MT-activities within the plan can be repeated several times, such as *Spring Tillages*, to control weeding or to increase infiltration capacity of the soil. The number of repetitions and the time between each repetition depend on the context and the triggering condition of the MT-activity. For MG11, the first spring tillage and each new occurrence of spring tillage must be separated by at least 30 days. Moreover each spring tillage requires at least 40 mm of rainfall on at least 5 ha of the vineyard (Table 2, descriptors 4 and 5). If these conditions are not satisfied, spring tillage is performed after at least 45 days have elapsed since previous tillage and no repetition is possible.

Other MT-activities are optional. This is the case for certain MT-activities that protect against downy and powdery mildew. In many grape-production systems, pesticides are regularly sprayed in spring and summer to control downy and/or powdery mildew, according to their respective pressure levels. This is the role of the iterated MT-activity *PesticidesSprayings* in Phase 3 of the MG11 plan. In addition, extra spraying may be needed when high levels of downy or powdery mildew are reached. They are represented in the plan by the optional MT-activities *ExtraAntiPowderyMildewSprayings* and *ExtraAntiDownyMildewSprayings*.

Within each phase of the plan, MT-activities are dynamically organised during the simulation according to their management options: opening and closing conditions, implementation mode, and priority degrees for resource allocation and execution. One characteristic of vineyard soil maintenance is the possibility of using different methods (e.g. tillage, chemical weeding, grass-cover mowing) for plots, for rows and inter-rows, and for inter-rows within the same plot. For example, chemical weeding and tillage in MG11 are performed on rows and inter-rows, respectively, of all plots. Management options are also used to represent the versatility of combining soil-maintenance methods through the specification of groups of plots that should be treated with the same combination of techniques. For other MT-activities, management options specify which method should be used on each plot when different methods are possible, such as chemical and/or manual shoot trunk removal. Most of the pesticide sprayings are applied on all the vine plots. There are however some exceptions that depend on varieties. For example, in MG11, the first anti-powdery mildew regular spraying only concern 4 varieties out of 12 (the earliest and most sensitive) and the extra sprayings only concerns one variety. These restrictions can be specified as management options.

## 4. The Simulation Process

The simulation is launched from the ‘main’ function of the simulator after reading files describing the vineyard, resource pools and their availability constraints, the plan and the management options representing the grape-production system to simulate. Dynamics of the simulated system depend on an agenda of events and the algorithmic functioning of the management system. In Dhivine, changes in the simulated system include the environment, grape phenological stages, resource availability, and the status and degree to which activities have been accomplished.

### 4.1. The agenda of events

According to design of the simulation engine, events trigger state changes in the simulated system and sustain them if they extend over a period. Some events are part of the knowledge in the field of study (e.g. grape phenology, triggering a mildew attack alert). Other events are predefined in the ontology of production systems because they have been recognised as generally useful (e.g. the *UpdateSituationEvent*, which updates the activity status from *sleeping* to *closed*, see subsection 4. 2). Some events are intentionally declared as input, while others are generated during simulation as consequences of the first and are inserted into the agenda. This is particularly the case when an event has a self-generation function whose role is to generate the same type of event and insert it into the agenda with an application-specific occurrence date. Whether events are intentionally declared beforehand or generated during simulation, they are stored in the agenda in ascending order of occurrence date. Until the agenda is empty or the simulation end date is reached, the simulation engine repeats the following actions: removal of the top event, advancing the simulation clock to the date the event occurs, and triggering the associated process.

For phenology dynamics, an initial event is intentionally programmed as simulation input. It initiates reading of a data file that contains the dates of stage change for each variety of interest (the varieties blueprint of Table 2, descriptors 9 and 10) and scheduling of an event that will prompt a stage change at the first date specified. When the simulation clock reaches the specified date, the stage change occurs, and a new stage change event is scheduled for the next date specified. This continues until the end date of the simulation.

A disease alert or a pest risk that could potentially affect the vineyard and justify spraying pesticides is modelled on the same principle as that used for stage changes. In both cases, events do not occur at regular intervals, but only on dates when changes in phenological stage or pest and disease pressure occur.

For weather data, a specific event is intentionally put in the agenda before the start of the simulation. It initiates a process that reads a line every simulated hour and gives the plots current values of weather variables (currently, only precipitation). This process is self-sustaining because the same event is automatically programmed to occur one hour later.

The state change of the operating system essentially concerns evolution of workers’ schedules (e.g. structure of the work week, legal limits to the number of work hours). This state is the main factor influencing availability of labour units outside vacation periods. A particular event is set to occur on the first hour of each day of the simulation period. For each labour unit, the associated process checks whether the current period remains relevant or the next period must be started.

### 4.2. Algorithmic functioning of the management system

The Dhivine management system model manager in Fig 1 is structured around the generic concept of a plan that specifies a flexible programme of actions. Each activity in the plan (and thus the plan) has a standard "life cycle" in which the status of the activity changes from *sleeping* to *waiting*, *open*, *underway* and, finally, *closed*. The overall control algorithm examines each activity whose status can change. The updating process for an activity checks whether the opening or closing conditions are satisfied and whether the constraints linking this activity to others would be satisfied if the change proceeded. Any activity with a valid change in status is updated, and the change is immediately propagated to the connected activities. When an MT-activity becomes *open*, a list of entities (plots, in Dhivine) to be processed is created using vineyard-specific expertise, and the MT-activity is expanded into a compound activity as a function of its implementation mode. From a functional viewpoint, checking whether the plan’s status should change is an instantaneous process triggered by an instance of the *UpdateStatusEvent* class in the DIESE framework. Only one updating event is intentionally scheduled to occur on the first day simulated, at a time specified as an input parameter. Subsequent updating events are self-generated until the end of the management period. The user specifies only the frequency of plan updates. The frequency is set as two hours in Dhivine. At each plan-status checkpoint, the algorithm identifies primitive activities with *open* or *underway* status to establish the set of tasks to initiate or continue. Any primitive activity with an unfulfilled feasibility condition is eliminated from the set.

The algorithm then attempts to allocate resources to the set of tasks (see [40] for detailed description of the algorithm). If global allocation is impossible, the set is broken down into smaller sets so that allocation can succeed. The allocation procedure considers resource-allocation priorities of MT-activities and their constituent primitive activities, the resource requirements of these activities, and the dynamics of resource availability. Finally, to determine which alternative candidate set of primitive activities to execute, the algorithm successively applies a set of preference criteria that considers execution priorities. The best activity set is then transmitted to the operating system for simultaneous execution of the activities. Simultaneous execution continues until the simulation clock triggers the event responsible for changing the plan status, causing a repeat of the above-described sequence.

## 5. Evaluation and Illustration of the Modelling Approach with a Case Study

We used Dhivine to simulate management practices of a previously surveyed vineyard. The study focused on analysis of pesticide spraying and tillage calendars across all plots. Focusing on these operations is justified by their strong impact on pesticide transfer in vineyards [41]. Pesticide spraying disseminates potentially contaminating active ingredients into the environment, while tillage strongly modifies infiltration capacity of the soil, with potentially large effects at catchment scales [42–44]. Determining the influence of vineyard-management strategies on the timing and location of pesticide spraying and tillage events is a major challenge to evaluating their hydrological impact.

### 5.1 Case study and simulation experiments

**Fig 4:**
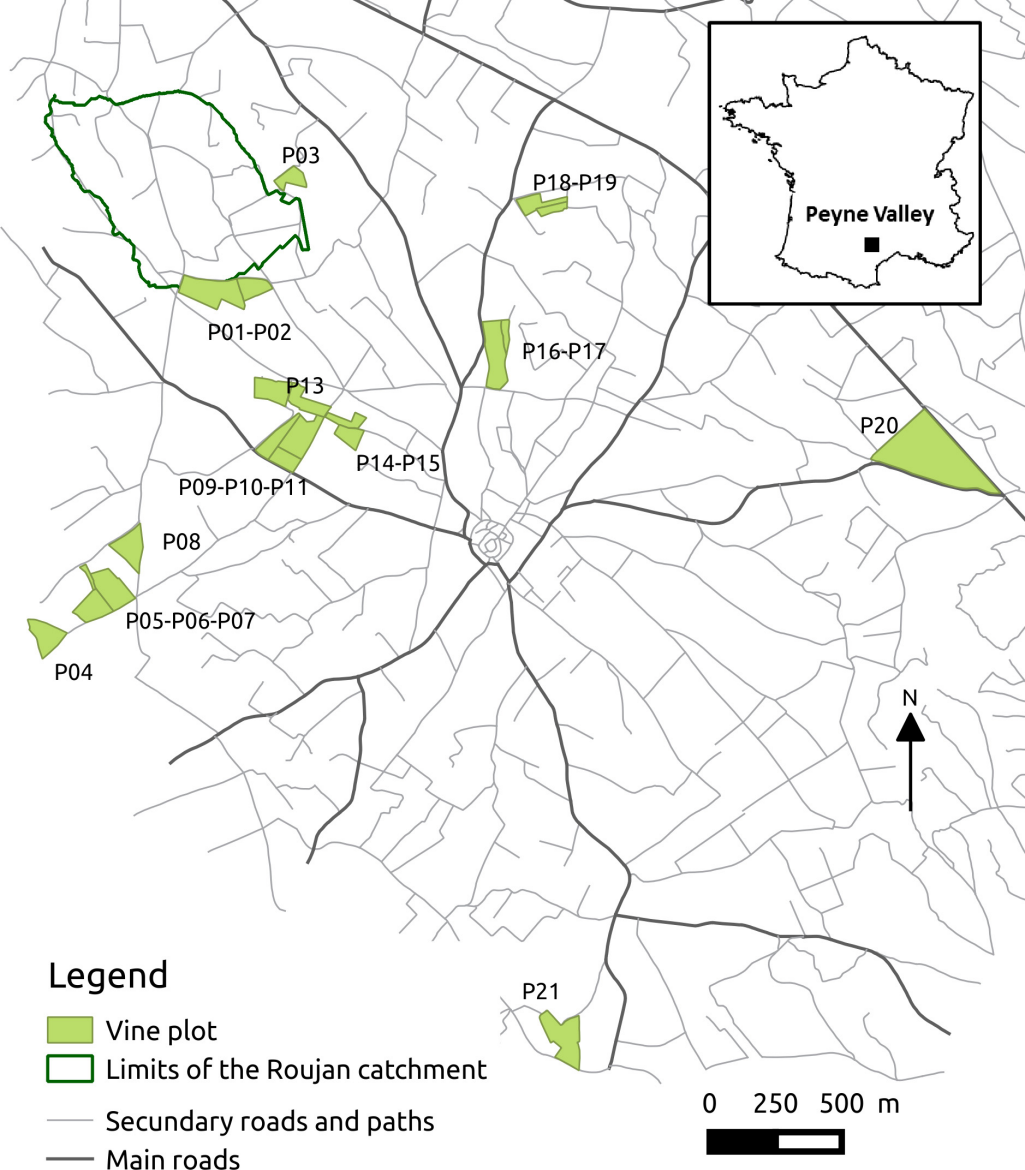
Location of the plots of the MG11 vineyard.

The simulation study focuses on the MG11 vineyard. (See Table 3 and Fig 4 for description of the vineyard, Tables 1 and 2 and Fig 3 for description of the equipment and labour requirements and management strategy). MG11 is located in the Peyne river valley in the Languedoc-Roussillon region of southern France, which is one of the largest wine-producing regions in the world. The region's climate is sub-humid Mediterranean. Annual rainfall is about 700 mm, but varies widely from year to year. There are sharp seasonal contrasts, with rainy autumns and springs, and hot, dry summers. In terms of physical characteristics, the Peyne river valley is part of the main vineyard landscape of southern Languedoc-Roussillon, which developed on top of Miocene marine and lacustrine sediments partially overlain by alluvial deposits [45]. Most of the valley has gentler landforms and is covered by vineyards. The soil pattern of the valley includes a great range of soil types that present contrasting characteristics in terms of soil texture. As shown in Table 3, the MG11 vineyard comprises 20 vines plots distributed between five types of soil [46]. Altitudes range from 60 m to 120 m. Most of the plots have a mean slope under 10%. In the case of steeper slope (plots P03, P16 and P18), terraces have been formed to facilitate machinery use. The diversity of the vine plots characteristics also concerns the varieties (12 different varieties), the vine density (from 3333 to 4444 vines/ha) and the pruning system (cane-trained or cordon-trained).

**Table 3:** Description of the plots of the MG11 vineyard.

Plot	Area (ha)	Variety	Row spacing (m)	Vine row spacing (m)	Vine density (vines/ha)	Pruning mode	Soil	Texture of the surface horizon (0-40/60 cm)	Mean slope (%)	Type of landscaping
P01	4.1	Muscat-Petit-Grain	2.5	0.9	4444	cordon-trained	Calcisol	Sandy clay loam	1.96	none
P02	1.5	Cabernet-Sauvignon	2.5	0.9	4444	cane-trained	Calcisol	Sandy clay loam	2.9	none
P03	1.5	Mourvedre	2.5	0.9	4444	cordon-trained	Calcisol	Silt loam	14.14	Terracing
P04	2.2	Syrah	2.5	1.2	3333	cordon-trained	Calcaric Cambisol	Silt loam	4.12	none
P05	2.0	Syrah	2.5	1.2	3333	cordon-trained	Calcisol	Silt loam	4.12	none
P06	0.3	Syrah	2.5	1.2	3333	cordon-trained	Calcisol	Silt loam	9.45	none
P07	2.8	Alicante-Bouschet	2.7	1	3704	cordon-trained	Calcisol	Silt loam	2.33	none
P08	2.3	Syrah	2.5	1.2	3333	cordon-trained	Calcisol	Sandy clay loam	2.88	none
P09	1.6	Syrah	2.5	0.9	4444	cordon-trained	Calcisol	Sandy clay loam	2.54	none
P10	1.3	Chardonnay	2.5	0.9	4444	cane-trained	Gleyic Calcisol	Sandy clay loam	2.17	none
P11	2.9	Cinsault	2.5	1.2	3333	cordon-trained	Calcisol	Clay loam	2.7	none
P12	3.8	Sauvignon	2.5	1	4000	cordon-trained	Calcisol	Sandy clay loam	4.13	none
P13	0.9	Merlot	2.5	1	4000	cordon-trained	Calcaric Cambisol	Silt loam	5.01	none
P14	1.2	Carignan	2	1.2	4167	cordon-trained	Calcisol	Silt loam	4.61	none
P15	3.5	Chardonnay	2.5	0.9	4444	cane-trained	Calcaric Cambisol	Silt loam	4.19	none
P16	0.6	Grenache	2	1.2	4167	cane-trained	Calcaric Cambisol	Silt loam	16.56	Terracing
P17	1.4	Petit Verdot	2.5	0.9	4444	cane-trained	Calcaric Cambisol	Silt loam	8.1	none
P18	0.6	Syrah	2.5	1	4000	cordon-trained	Calcisol	Silt loam	13.81	Terracing
P19	13.2	Cabernet-Sauvignon	2.5	1	4000	cordon-trained	Fluvisols	Silt loam	1.33	none
P20	3.8	Carignan	2	1.2	4167	cordon-trained	Luvisol	Sandy clay loam	1.15	none

As seen in Fig 4, all MG11’s plots are located within 5 km of one another. Among them, plot P01 that is located in the Roujan catchment (91 ha) has been monitored for runoff flows over several years. The tillage, chemical weeding, and anti-mildew spraying practices on P01 were recorded by the vineyard manager over two successive cropping cycles (2004–2005, 2005–2006). Weekly observations provided chemical-weeding and tillage dates of P01 for an additional cycle (2006–2007).

The simulation experiments aimed to (i) validate the simulated chronology of tillage, chemical weeding, and pesticide spraying by comparing it to the observed chronology and (ii) compare effects of two changes in management options on pesticide-spraying and tillage scheduling. The first change modified execution priorities to provide more balanced allocation of labour between trellising and other competing MT-activities (i.e. spring tillage, pesticide spraying, shoot removal and trimming), a strategy observed in other surveyed vineyards. With this modification, trellising can be performed simultaneously with other activities. In the observed strategy, trellising had a higher execution priority than the competing MT-activities, except for pesticide spraying. For the manager, performing several activities simultaneously or giving priority to trellising is a matter of choosing between not falling behind in an MT-activity or reducing the risk of branches breaking due to wind. The second modification changed the method of trunk shoot removal from chemical-based to manual-based operations to adopt a more environmentally friendly practice. This alternative requires more labour and significantly reduces the unitary speed of the activity (0,06 ha/h/person instead of 0,83 ha/h/person).

**Fig 5:**
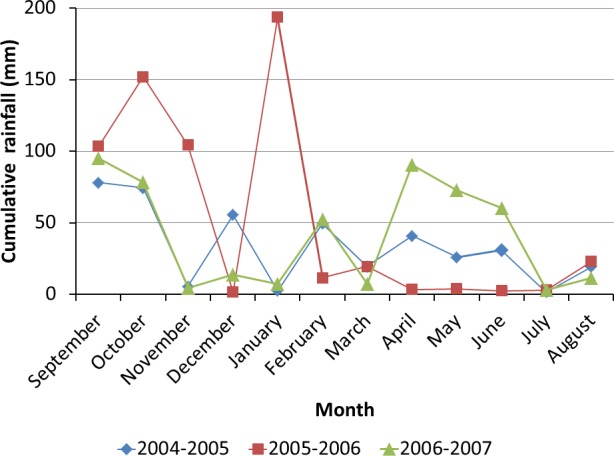
Distribution of monthly rain during the three agricultural cycles considered

Rainfall data (Fig 5) were obtained from the rainfall station on the Roujan catchment (43°30’N, 3°19’E, altitude: 76 m). The three cropping cycles simulated cover a wide range of variation in annual rainfall, (404, 623 and 498 mm). Phenological stages were observed during the three crop cycles in a nearby experimental field. Downy-mildew and powdery-mildew alert levels were taken from the Performance Vigne agricultural warning service. Downy mildew pressure was observed only during the 2004–2005 cycle.

For each plot, the type of soil and the texture were obtained from the soil map of the Peyne valley [45] and named according to the WRB classification [46]. The mean slope was obtained from DEM (digital elevation model from IGN). The other data were observed or collected by survey.

### 5.2 Results and discussion

#### 5.2.1. Assessment of Dhivine predictions

**Fig 6:**
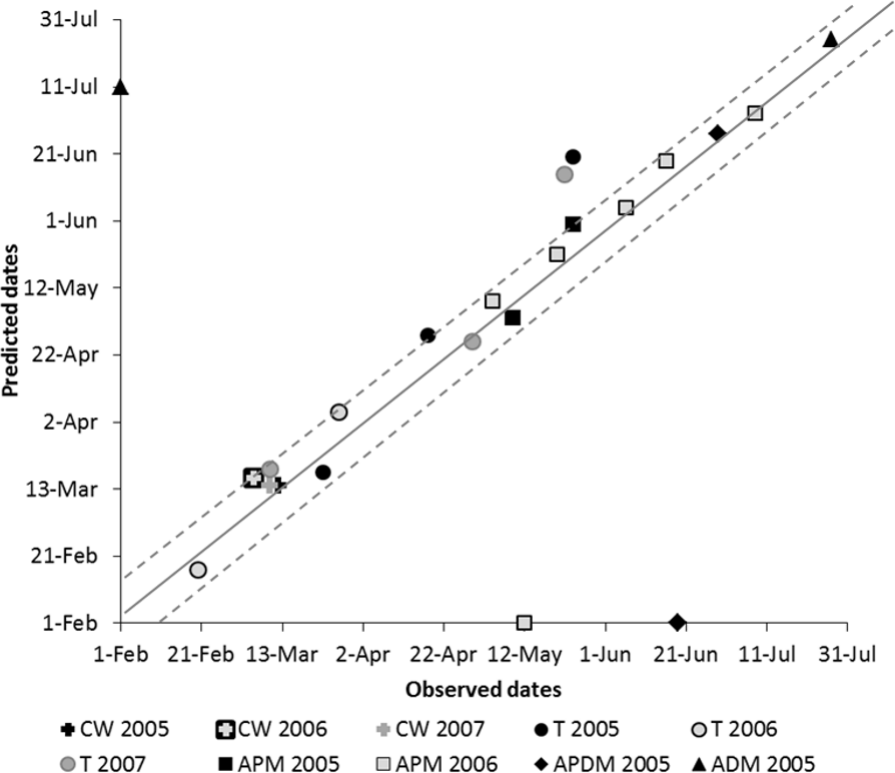
Observed and predicted dates of cultivation operations on the Roujan plot of the MG11 vineyard from January to August of 2005–2007. Operations: CW = chemical weeding; T = tillage; APM = anti-powdery mildew spraying; ADM = anti-downy mildew spraying; APDM = anti-powdery and anti-downy mildew spraying. Dashed lines indicate ± 10 days around observed dates. The two points on the x-axis correspond to observed sprayings that were not simulated, while the point on the y-axis corresponds to a predicted spraying that was not observed.

The predicted chronologies of chemical weeding, late-winter and spring tillage and pesticide spraying on the Roujan plot for the 2004–2005, 2005–2006 and 2006–2007 cycles are shown in Fig 6. Predictions were the total number of occurrences of each operation and their implementation dates. For tillage operations, predictions equalled the observed number of occurrences (a function of weather variability), with three occurrences each in 2004–2005 and 2006–2007 and two in 2005–2006 (Fig 6). The latter cycle had a wet winter that prompted early resumption of tillage and a dry spring that did not necessitate tillage. Weather characteristics of the 2004–2005 and 2006–2007 cycles were opposite (Fig 5): dry winters justified delayed tillage, and rainy springs required an additional tillage.

The occurrence of anti-powdery and anti-downy mildew spraying was also satisfactorily predicted: 9 of the 12 observed sprayings (75%) were predicted (Fig 6). Dhivine also accurately predicted the target (powdery and/or downy mildew) as a function of disease pressure: anti-powdery and anti-downy mildew spraying during spring and summer of 2005 and no anti-downy mildew spraying during those of 2006.

Excluding the three poorly predicted sprayings, all operation dates were predicted with a mean error of 7 days, and 90% were predicted with an error of no more than 10 days. Prediction of spraying was more accurate (mean error of 4 days) than that of chemical weeding (mean error of 5 days) and tillage (mean error of 11 days).

These results indicate that Dhivine realistically represented vineyard management of MG11, since it satisfactorily predicted the number and timing of operations at the plot scale. As for any model the quality of simulation results depends on the quality of inputs. Dhivine is sensitive to the regional agricultural warnings that do not always accurately represent the pest- and disease-pressure levels of a particular vineyard. This factor is uncontrollable. The accuracy of the model can certainly be improved by refining the conditions that determines tillage dates and the number of anti-mildew sprayings. The concerned imperfections may come from inappropriate opening predicates and feasibility conditions, improper evaluation of nominal speeds of operations (particularly in the case of manual operations), excluded maintenance activities (e.g. of trellis systems or ditches), inaccurate representation of cyclical adjustments of the workforce, and failure to include adjustments of the nominal plan of activities between two crop cycles. Currently, the tillage opening predicates, soil trafficability conditions and workability conditions are based on thresholds of cumulative rainfall that are therefore the same for all plots; their differences in terms of type of soil and texture are not presently taken into account in the MG11 example. Another potential improvement is considering other activities that may consume significant resources and affect scheduling. Regarding pesticide sprayings, the model finely represents how vine growers take into account the diversity of grape varieties to schedule pesticide sprayings. This is done through (i) explicit spraying restrictions that are varieties-dependent (ii) opening and closing predicates that are defined with respect to the phenological stages of standard varieties (the early and late varieties blueprint in Table 2, descriptors 8 and 9). Presently the model does not account for the history of pest and disease infestations on the plots and does not simulate a related modulation of spraying.

#### 5.2.2. Impacts of work-organisation changes

**Fig 7:**
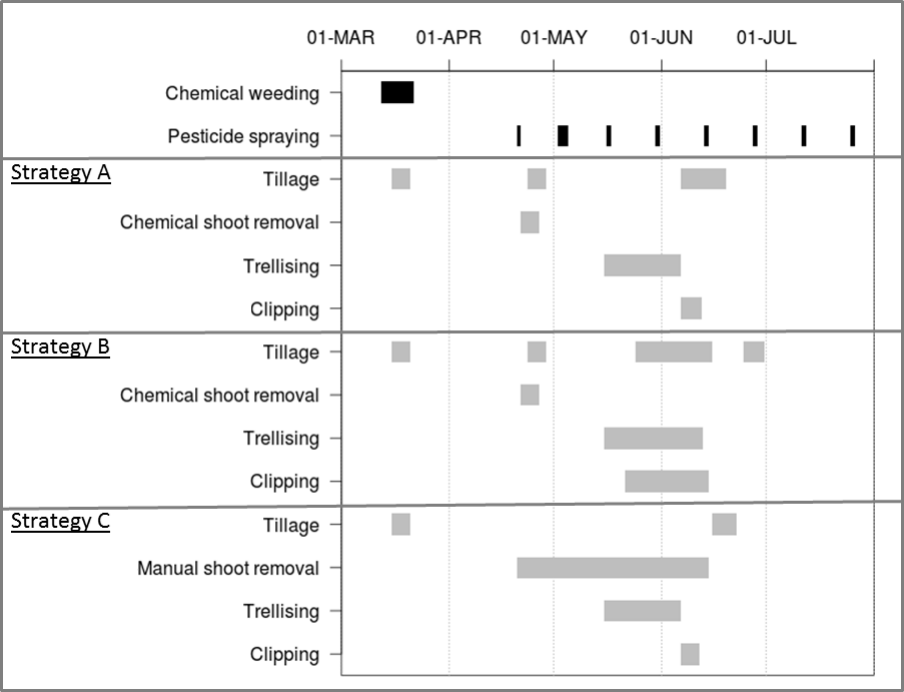
Timing of predicted MT- activities from March to July 2007 for three strategies. Strategy A = the current management strategy, Strategy B = based on A with a change in execution priorities, Strategy C = based on A with change in shoot-removal method at the vineyard scale. Horizontal bars indicate the durations of activities from beginning to ending dates. Activities in black on the top of the figure are common to all three strategies, not having been influenced by changes.

Impacts of changes in the two management options occurred in spring and early summer. Options were evaluated across the vineyard for the 2006–2007 cycle and compared to the original options. Fig 7 shows timing of the MT-activities performed during spring and early summer for the original management options (strategy A) and the two new options (strategies B and C, respectively). Chemical weeding and pesticide spraying were not affected by the changes due to the former being performed in late winter and the latter having a high execution priority (Fig 7). In contrast, the changes influenced the timing and durations of tillage, shoot trunk removal, trellising and trimming and the number of tillages (Fig 7B and 7C). Modifying execution priorities to perform trellising at the same time as other activities made an additional tillage possible (Fig 7B). Conversely, changing trunk shoot removal from a chemical to a manual operation removed one tillage because the latter required more time (Fig 7C).

These predictions illustrate the strong impact that management options have on the calendar of low-priority MT-activities, such as tilling in MG11. They indicate that Dhivine is suitable for predicting the impact that different management strategies have on crop management sequences by simply varying management options. The impact of more radical changes could also be tested, such as transition to an organic system or financial difficulties that decrease resource capacity.

## 6. Conclusion and Future Work

Dhivine realistically simulates grape production at the farm scale and the management processes responsible for dynamic scheduling of activities. Its originality lies in expressing the diversity of management strategies. The model is well-adapted for studying centralised management that focuses on the timing and contextualisation of activities, which must be known to accurately assess environmental impacts of cropping systems. Our modelling approach enhances traditional farm-analysis tools by providing a holistic and process-oriented view of management operations.

The complexity of the farmer’s management task is not solely due to the number of components or possible states of the system. It is also due to the dynamic context-dependent behaviour of the components, which arises from their interactions over time and depends on uncontrollable driving factors, such as weather. The dynamic complexity is related to human difficulties in consistently dealing with multiple and delayed consequences of activities. Much of the information about biophysical-system functioning and the cognitive processes involved in production management is based on farmers’ tacit knowledge. Dhivine-like models can capture some of this subjective and context-specific knowledge and thus promote scientific investigation of production management. Both research and practice benefit from the ability to make this knowledge explicit and usable for formal modelling and learning. Researchers can build more realistic and insightful models, and farmers can increase their awareness of and skills in organisational and management issues

However, an unavoidable trade-off exists between the realism, precision and intelligibility of models developed with this approach. It is infeasible to model all activities and accurately capture the beliefs, intentions, attitudes and constraints of those managing a production system. It seems impossible to predefine all possible contingencies that may affect a manager. More fundamentally, understanding of the cognitive processes that production managers use to make decisions remains limited. For example, Dhivine does not represent mechanisms such as goal-based reasoning or uncertainty processing. It also does not account for workers’ relative autonomy in decision-making or the interactions that might lead to interpersonal coordination opportunities or conflicts. Therefore, this modelling approach can only predict patterns rather than provide the precise predictions possible for non-complex phenomena. Dhivine is not intended to replicate the decision process of a particular manager, which seems beyond current capabilities of cognitive and decision science. Instead, it aims to determine spatial and temporal patterns of operations produced by implementing a user-designed management strategy (possibly inspired by those observed) and whether these patterns are qualitatively consistent with the expectations of experts (e.g. researchers, vineyard managers, advisers). To assess the model’s consistency, it is necessary to analyse the overall soundness of simulations of a single strategy under different scenarios of external drivers and various resource capacities. The analysis should simulate several strategies to cover a range of predictable behaviours with known inter-relationships. This validation process has yet to be deeply explored and formalised for Dhivine.

Dhivine benefits from the versatility of the DIESE modelling framework, whose large scope of applicability [32, 35–38] stems from the genericity of its underlying ontology of production system. Dhivine introduced the concept of an MT-activity, which increased the pattern of work organisation that can be represented and processed. Improving the realism of simulated management behaviour is one way to approach the issues found in practice. The approach is appropriate for analysing the selection and prioritisation of activities that are organised in a flexible plan. Dhivine is the largest DIESE-based management model in terms of the number of operations and resource items manipulated. Despite the diverse applications of DIESE, building such complex models remains a highly specialised skill. There is still much to learn about how to build models from elicited knowledge, debug them and effectively use them.

Development of Dhivine was motivated by the need to assess environmental impacts of grape-production management practices, particularly pesticide spraying and tillage. In this study, the model was used to reproduce calendars and locations of tilling and pesticide spraying on a single vineyard. This is not sufficient to address the original issue, because the consequences of practices often transcend farm boundaries, and environmental impacts result from a complex interplay between the management of vineyards in the same catchment. The next step is to apply Dhivine to a set of vineyards managed by different managers, each having her/his own strategy and resource capacity. Environmental assessment will require combining Dhivine with an eco-hydrological model at the catchment scale to simulate hydrological dynamics (e.g. water balance, pollutant transport, erosion, soil-surface conditions) influenced by and driving management operations of the managers. Thus, coupling of Dhivine and the eco-hydrological model MHYDAS [43] is the focus of current research.
